# The Chinese Herbal Decoction Danggui Buxue Tang Inhibits Angiogenesis in a Rat Model of Liver Fibrosis

**DOI:** 10.1155/2012/284963

**Published:** 2012-07-29

**Authors:** Jing Lv, Zhimin Zhao, Yuan Chen, Qinglan Wang, Yanyan Tao, Li Yang, Tai-Ping Fan, Chenghai Liu

**Affiliations:** ^1^Institute of Liver Diseases, ShuGuang Hospital, Shanghai University of Traditional Chinese Medicine, Shanghai 201203, China; ^2^The MOE Key Laboratory for Standardization of Chinese Medicines and the SATCM Key Laboratory for New Resources and Quality Evaluation of Chinese Medicines, Institute of Chinese Materia Medica, Shanghai University of Traditional Chinese Medicine, Shanghai 201210, China; ^3^Angiogenesis & Chinese Medicine Laboratory, Department of Pharmacology, University of Cambridge, Cambridge CB2 1PD, UK; ^4^E-Institute of TCM Internal Medicine, Shanghai Municipal Education Commission, Shanghai 201203, China; ^5^Shanghai Key Laboratory of Traditional Chinese Clinical Medicine, Shanghai 201203, China

## Abstract

In this study, we investigated the anti-angiogenic effect of the Chinese herbal decoction Danggui Buxue Tang (DBT; *Radix Astragali* and *Radix Angelicae sinensis* in 5 : 1 ratio) in a rat model of liver fibrosis, in order to elucidate its mechanisms of action against liver fibrosis. Liver fibrosis was induced with CCl_4_ and high-fat food for 6 weeks, and the rats were treated with oral doses of DBT (6 g raw herbs/kg/d) and N-Acetyl-L-cysteine (NAC; 0.1 g/kg/d). The results showed that both DBT and NAC attenuated liver fibrosis and neo-angiogenesis. Furthermore, DBT and NAC improved SOD activity but decreased MDA content and 8-OH-dG in fibrotic livers, with DBT being more effective than NAC. DBT decreased the expression of VEGF, Ang1 and TGF-**β**1 and their signaling mediators, whereas NAC had no effect on VEGF and VEGFR2 expression. Both DBT and NAC reduced HIF-1**α** gene and protein expression in fibrotic livers, with DBT being more effective. These data clearly demonstrate that the anti-fibrotic properties of DBT are related to its ability to inhibit angiogenesis and its anti-angiogenic mechanisms are associated with improving oxidative stress, regulating the expression and signaling of angiogenic factors, and especially modulating HIF-1**α** in fibrotic livers.

## 1. Introduction

Angiogenesis is a hypoxia-driven and growth factor-dependent process that leads to the formation of neovasculature from preexisting blood vessels. Experimental and clinical studies have unequivocally shown that pathological angiogenesis, irrespective of etiology, plays a key role in the fibrogenic progression of chronic liver diseases [1–3], and the inhibition of pathological angiogenesis in liver not only can stop liver cancer development, but also regress or reverse liver fibrosis [4, 5]. 

Danggui Buxue Tang (DBT), an ancient traditional Chinese herbal formula composed of Huangqi (*Radix Astragali*) and Danggui (*Radix Angelica sinensis*) with a weight ratio of 5 : 1, has wide pharmacological actions, including regulation of immune functions and protection against liver injuries [6]. Although there are no reports concerning the effect of DBT on liver cirrhosis, several studies have reported antifibrotic effects for its components. For example, the combination of *Astragali* and *Angelicae sinensis* significantly inhibited the progression of renal fibrosis. This treatment led to a decrease in histologic damage, type III and IV collagen expression, fibronectin, and laminin in a rat model of chronic puromycin-induced nephrosis [7]. *Astragali* significantly attenuated liver tissue collagen and hydroxyproline (Hyp) content in a rat model of liver fibrosis induced by albumin immune complex [8]. In a rat model of pulmonary fibrosis induced by intratracheal instillation of bleomycin, *Angelica sinensis* ameliorated fibrosis by inhibiting thromboxane B_2_ level and transforming growth factor-*β*1 (TGF-*β*1) expression [9]. 

Our previous study found that DBT was able to ameliorate liver fibrosis induced by carbon tetrachloride (CCl_4_) in rats, with the best results seen with a 5 : 1 ratio of *Radix Astragali *and* Radix Angelicae sinensis* [10]. In a subsequent study, we showed that this protective effect of DBT was associated with the prevention of lipid peroxidation and the inhibition of matrix metalloproteinases 2/9 (MMP 2/9) activities in fibrotic livers [11]. 

In the present study, we observed the effects of DBT on angiogenesis in fibrotic livers, with the antioxidant N-Acetyl-L-cysteine (NAC) as a positive control drug. To test the hypothesis that the antifibrotic properties of DBT are related to its ability to inhibit angiogenesis, we also analyzed its effects on oxidative stress injury, expression of VEGF, TGF-*β*1, and Ang1, expression of VEGF-R2, TGF*β*-R1/2 and Tie2 receptors, and ERK phosphorylation, in particular HIF-1*α* expression in the fibrotic liver. Collectively, our data demonstrate that improving oxidative stress, regulating angiogenic factorsexpression and signaling, and especially modulating the gene and protein expression of HIF-1*α*. 

## 2. Materials and Methods

### 2.1. Reagents

CCl_4_, olive oil and N-Acetyl-L-cysteine (NAC) were purchased from Shanghai National Chemicals Co., Ltd. (Shanghai, China). Bovine serum albumin (BSA) was purchased from Sino-American Biotechnology Co. (Henan, China). The primary antibodies used in this study are listed in Table 1. Nitrocellulose membrane (Hybond-C, optimized for protein transfer) was purchased from Amersham Biosciences UK, Ltd. (Buckinghamshire, UK). Horseradish peroxidase-labeled goat anti-mouse antibody and goat anti-rabbit antibody were obtained from Santa Cruz Biotechnology (California, USA). Cy3-labeled goat anti-rabbit IgG (H+L), Cy3-labeled donkey anti-goat IgG (H+L), and 2-(4-Amidinophenyl)-6-indolecarbamidine dihydrochloride (DAPI) were provided by the Beyotime Institute of Biotechnology (Jiangsu, China). The BCA Protein Assay Kit and the SuperSignal West Pico Chemiluminescent Substrate (ECL) were obtained from Pierce Chemical Company (Rockford, USA). TRIzol was obtained from Invitrogen (California, USA). The First-Strand cDNA Synthesis Kit (K1622) was purchased from Fermentas (St. Leon-Roth, Germany). The SYBR Green Real Time PCR Kit (DRR041A) was from TakaRa Biotechnology Co., Ltd (Dalian, China). Superoxide dismutase (SOD), malondialdehyde (MDA), and 8-hydroxy-deoxyguanosine (8-OH-dG) were obtained from Nanjing Jiancheng Bioengineering Institute (Nanjing, China).

### 2.2. Preparation of Danggui Buxue Decoction

DBT consists of *Radix Astragali* and *Radix Angelica sinensis* in a 5 : 1 ratio. The herbs originated from Gansu province, China. Slices of the herbs were purchased from Shanghai Huayu Chinese Herbs Co., Ltd. The medicinal herbs were extracted twice. Radix Astragali (1000 g) and *Radix Angelica sinensis* (200 g) were first boiled together in 6x volume of water for 1 h, and then the residue from first extraction was boiled in 8x volume of water for 1.5 h. Finally, the filtered solutions were combined and concentrated into the resulting aqueous extracts containing 0.9 g/mL raw herbs. The quantitative analyses of active compounds were verified by Professor Li. Yang (Table 2).

### 2.3. Animal Models of Liver Fibrosis and Drug Treatment

Fifty-four male Wistar rats (SCXK [Shanghai] 2007-005) were obtained from the Shanghai Laboratory Animal Center of the Chinese Academy of Sciences. All animal protocols were carried out in accordance with ethical guidelines, and animals had free access to chow and water throughout the experiments. Liver fibrosis was induced by subcutaneous injection of CCl_4_ and the administration of food with a high lipid content and lower protein content [12]. Briefly, the rats received a single injection of 100% CCl_4_ at 5 mL/kg and then 3 mL/kg of 40% CCl_4_ dissolved in olive oil twice every week for 6 weeks. These rats were pair-fed with a high lipid and low protein diet containing 79.5% corn flour, 20% lard, and 0.5% cholesterol for the first 2 weeks, then with pure corn flour feeds for the following 4 weeks. Rats in the normal group (*n* = 8) did not receive CCl_4_ treatment and were fed a normal diet.

The model rats were randomly divided into three groups: model (*n* = 12), DBT (*n* = 12), and NAC (*n* = 12). The rats in the DBT group received intragastric administrations of DBT at 6 g (raw herbs)/kg/d. Rats in the NAC group received intragastric administrations of NAC at 0.1 g/kg/d. Both drug treatment duration is 6 weeks, which means from the beginning of intoxication to the end of experiment, and both dosages are equivalent to 10 times the clinical dosage for a 60 kg adult. Rats in the normal and model groups received the same volume (10 mL/kg) of normal saline. 

### 2.4. Pathological Examination

Liver specimens were preserved in 4% formaldehyde and dehydrated in a graded alcohol series. The specimens were then embedded in paraffin blocks, cut into 4 *μ*m thick sections and placed on glass slides. Sections were then stained with Sirius red.

### 2.5. Hepatic Hyp Content Examination

Hepatic Hyp content was measured with a modified version of the method developed by Jamall et al. [13]. Briefly, 100 mg of liver samples were homogenized and hydrolyzed in 12 M HCl at 110°C for 18 h. After filtration of the hydrolysate through filtration paper, chloramine T was added to a final concentration of 2.5 mM for 10 min at room temperature. The mixture was then treated with 25% (w/v) p-dimethylaminobenzaldehyde and 27.3% (v/v) perchloric acid in isopropanol (Ehrlich's reagent solution) and incubated at 50°C for 90 min. After cooling to room temperature, the samples were examined at 558 nm against a reagent blank that contained the complete system without tissue. The concentration of Hyp in each sample was determined from a standard curve, which was generated from a serial of known quantities of Hyp from 0.2 to 1.6 *μ*g Hyp (Peptide Co. Japan). The Hyp content is expressed as *μ*g/g of liver wet weight.

### 2.6. Immunohistochemistry

4 *μ*m thick sections were used for immunohistochemical examinations. After deparaffinization and dehydration, microwave antigen retrieval was performed for 5 min prior to peroxidase quenching with 3% H_2_O_2_ in PBS for 15 min. Consequently, the sections were preblocked with 5% bovine serum albumin for 30 min. Slides were incubated at 4°C with primary antibodies (Table 1) at 37°C for 70 min and then with biotinylated secondary antibodies for 45 min. They were then developed with DAB for 3 min and finally counterstained with hematoxylin. For the negative controls, the primary antibody was replaced with PBS.

### 2.7. Immunofluorescence

Frozen liver tissue slices (7 *μ*m thick) were fixed with cold acetone for 10 min and dried in air for 30 min. The slices were rinsed with PBS for 3 × 5 min and blocked with 0.2% BSA for 1 h at 37°C before being incubated with the specific primary antibodies (Table 1). To visualize the primary antibodies, the slices were stained with cy3-labeled secondary antibodies for 1 h at 37°C. After nuclear staining with DAPI (1 : 1000; Beyotime) for 1 min, the slices were observed using immunofluorescence microscopy (OLYMPUS ZX70). Negative control staining was performed using PBS instead of the primary antibody. Microvessel density was determined in the tissue as the mean number of labeled vessel sections in 5 successive high-magnification fields (×400) with IPP software. The results are expressed as the means ± SD.

### 2.8. Western Blotting Analysis

Liver tissues were homogenized in RIPA lysis buffer (150 mM NaCl, 1% Nonidet P-40, 0.1% SDS, 50 mM Tris-HCl pH 7.4, 1 mM EDTA, 1 mM PMSF, and 1×Roche complete mini protease inhibitor cocktail). The supernatants were collected by centrifugation at 10,000 ×g at 4°C for 15 min. Protein concentration was determined using a BCA Protein Assay Kit. Equal amounts of protein were separated by 10% SDS gel electrophoresis (SDS-PAGE) under denaturing and nonreducing conditions and then transferred to a nitrocellulose membrane. The membrane was blocked with 5% nonfat milk in TBST at room temperature for 1 h and then incubated with primary antibody (Table 1) at 4°C overnight. After washing in TBST, the blots were incubated with a horseradish-coupled secondary antibody. The signals were visualized using the enhancement system (ECL).

### 2.9. Fluorescence Western Blot

The fluorescence western blot protocol is the same as the general western blot protocol until the membrane transformation step. The membranes were blocked in blocking buffer (Odyssey, LI-COR, USA) for 1 h at room temperature followed by incubation with primary antibodies (Table 1) at 4°C overnight. After washing in PBS, the blots were incubated with the second antibody (Donkey anti-Rabbit IRDye 680 antibody; 1 : 10000; Odyssey) for 1 h at room temperature. After washing, the densities of immunoreactive bands were quantified and corrected for GAPDH signal with Li-Cor Odyssey 2.1 software (LI-COR, USA).

### 2.10. RNA Extraction and Quantitative Real-Time PCR

Total RNA was isolated from the liver tissues with TRIzol according to the manufacturer's instructions. RNA purity was determined spectrophotometrically, and its integrity was verified by agarose gel electrophoresis. First-Strand cDNA was synthesized by reverse transcribing 4 *μ*g of total RNA in a final reaction volume of 20 *μ*L using a First-Strand cDNA Synthesis Kit according to the manufacturer's instructions. Primer oligonucleotide sequences specific for real-time PCR are shown in Table 3 and were designed and synthesized by Sangon Biotech Inc. (Shanghai, China). The PCR mixtures contained 1 *μ*L cDNA, 10 *μ*L SYBR Premix Ex Tq 2X, and 0.25 *μ*mol/L forward and reverse primers in a final volume of 20 *μ*L. Triplicate reactions were performed with a Rcorbett 6.0 system (Rotor-Gene 3000), starting with a polymerase activation step for 10 s at 95°C, followed by 40 cycles of 5 s at 95°C and 20 s at 60°C. Fluorescence data were acquired after each cycle. The absence of primer dimers and unspecific products was verified after every run by melting curve analysis (72 to 95°C) and agarose gel electrophoresis.

### 2.11. Hepatic Oxidative Injury Examination

SOD, MDA and 8-OH-dG levels in the liver homogenates were quantified using commercially available kits according to the manufacturer's instructions (Nanjing Jiancheng Co. Ltd.). Results were normalized to the total amount of protein measured by bicinchoninic acid (BCA).

### 2.12. Statistical Analysis

Statistical tests were performed using SPSS software version 12.0. Differences between two groups were analyzed by the SNK-*q*' test. *P* values lower than 0.05 were considered statistically significant.

## 3. Results 

### 3.1. DBT and NAC Ameliorated Liver Fibrosis Induced by CCl_4_

**Figure 1:**
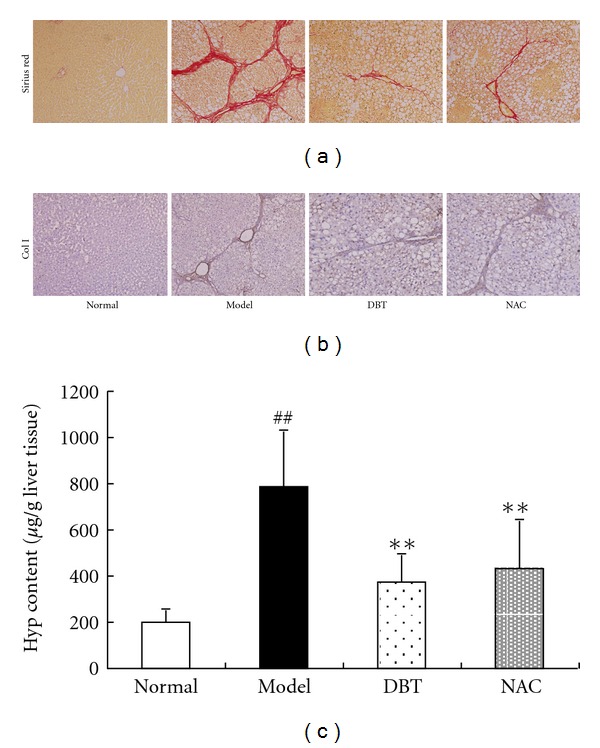
Effects of DBT and NAC on liver fibrosis induced by CCl_4_. (a) Sirius red staining of collagen deposition in liver fibrosis. CCl_4_ induced an increase in liver collagen. DBT and NAC decreased the collagen deposition in the fibrotic liver tissue. (b) Immunohistochemical staining of type I collagen protein expression in liver tissue (DAB). The model group had much stronger and more extensive positive staining among fibrotic septa compared with the normal group. DBT and NAC significantly decreased the amount of positive staining. (c) Liver Hyp content was determined using Jamall's method. The Hyp content was increased significantly in the model group compared with the normal group. DBT and NAC treatment significantly decreased liver Hyp content. ^##^ *P* < 0.01 versus normal group, **P < 0.01 versus model group.

The model rats had increased collagen deposition in the liver, which formed fibrous septa and cirrhotic nodules or pseudolobules. The doses of DBT and NAC used in this study were calculated according to their usage in patients with liver cirrhosis. Figures 1(a) and 1(b) show that DBT- and NAC-treated rats had much less liver collagen accumulation compared to the model control. This was confirmed with Hyp content as a specific marker for collagen synthesis. Compared to normal rats, Hyp content was increased significantly in model rats and significantly decreased by DBT and NAC treatment (*P* < 0.01; Figure 1(c)). 

### 3.2. Effect of DBT and NAC on Hepatic Stellate Cell (HSC) Activation

**Figure 2:**
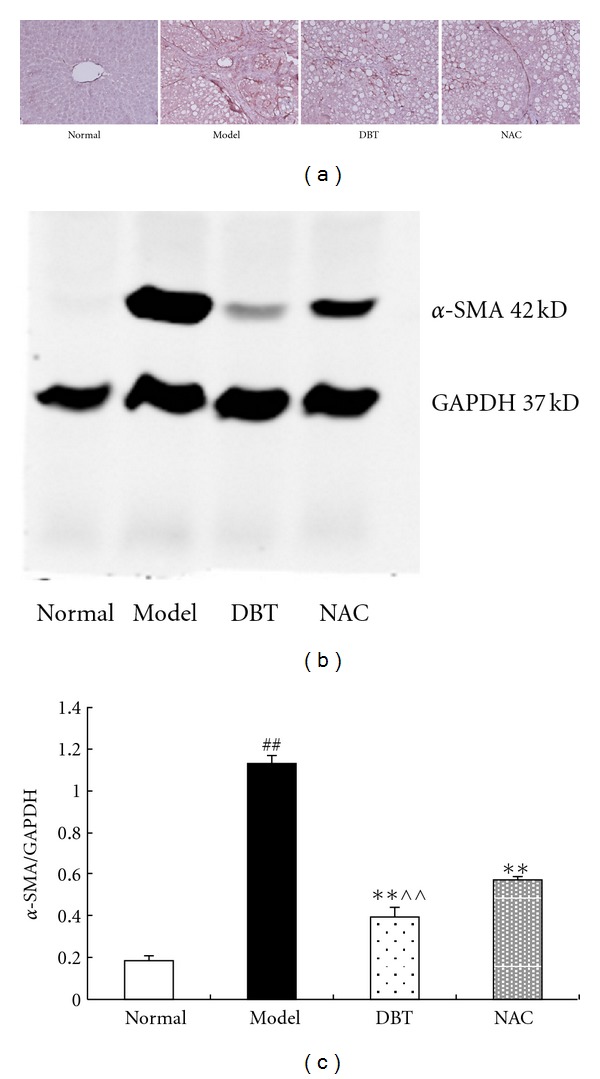
Effect of DBT and NAC on *α*-SMA protein expression in CCl_4_-induced fibrotic liver tissue in rats. (a) Immunohistochemical staining, DAB × 200. Normal liver tissue had little positive staining. Tissue from the model group had much stronger and more extensive positive staining among fibrotic septa and in the sinusoidal area. DBT and NAC significantly decreased the amount of positive staining. (b) A representative western blot. Consistent with the immunohistochemistry findings, the semi-quantitative value of the *α*-SMA western blot was significantly increased in fibrotic livers. Compared to the model control group, DBT significantly decreased *α*-SMA expression, and the effect of DBT was more pronounced than that of NAC. (c) A histogram plot showing the densitometric analysis corresponding to the mean ± SD of three independent experiments. ^##^ *P* < 0.01 versus normal group, **P < 0.01 versus model group, ^∧∧^ *P* < 0.01 versus NAC group.

*α*-SMA is a marker of HSC activation. We measured the expression of *α*-SMA by immunohistochemical staining and western blot analysis. CCl_4_ treatment caused a significant increase in the expression of *α*-SMA positive HSCs around damaged hepatocytes and fibrotic bands compared to the normal group. In DBT- and NAC-treated animals, the number of *α*-SMA-positive HSCs was significantly reduced. Western blot analysis revealed that the *α*-SMA expression in model rats was almost 5 times higher than that in normal rats. Treatment with DBT and NAC resulted in significant reductions. In addition, there was a significant difference between the DBT and NAC group (*P* < 0.01; Figures 2(a), 2(b) and 2(c)); DBT was more effective.

### 3.3. Effect of DBT and NAC on Tissue Angiogenesis

**Figure 3:**
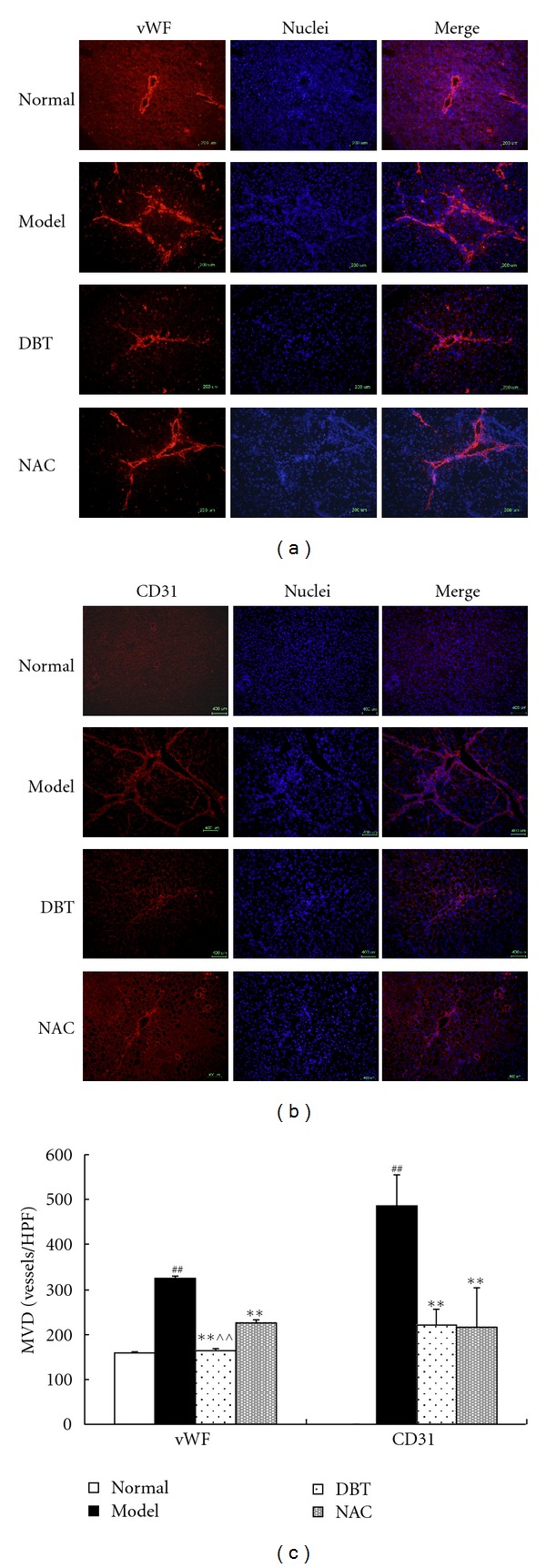
Effect of DBT and NAC on vWF and CD31 expression in CCl_4_-induced fibrotic liver tissue in rats, (a, b) Microvessel density in different groups. Immunofluorescence for vWF and CD31 was performed to analyze microvessel growth. In normal livers, the expression of vWF was limited to large vessels located in the periportal zone with no expression along the sinusoids, and no CD31 expression was observed. In CCl_4_-treated livers, increased vWF/CD31-positive microvessels were seen primarily in fibrotic areas. DBT- and NAC-treated livers showed reduced amounts of vWF/CD31-positive microvessels, which were also concentrated in fibrotic areas. A significant difference in vWF expression was seen between DBT- and NAC-treated groups. (c) A bar graph representation of microvessel density reported as the mean number of labeled vessel sections in 5 successive high-magnification fields (×400). The results are expressed as mean ± SD for three separate experiments. ^##^ *P* < 0.01 versus normal group, **P < 0.01 versus model group, ^∧∧^ *P* < 0.01 versus NAC group.

Immunofluorescence for von Willebrand factor (vWF) and platelet/endothelial cell adhesion molecule-1 (PECAM-1, also referred to as CD31) was performed to analyze microvessel growth. In normal livers, vWF is expressed in the great vessels of the portal tract and central veins but not along sinusoids, and CD31 is rarely expressed. In CCl_4_-treated livers, we observed vWF/CD31-positive microvessels located in fibrotic areas surrounding larger vessels, as well as in emerging fibrotic septa. As illustrated in Figure 3(c), DBT and NAC reduced the number of vWF/CD31-positive microvessels, which were also concentrated in fibrotic areas. DBT was more effective than NAC in reducing vWF expression (*P* < 0.01). 

### 3.4. Effect of DBT and NAC on Hypoxia Inducible Factor (HIF)-1*α*

**Figure 4:**
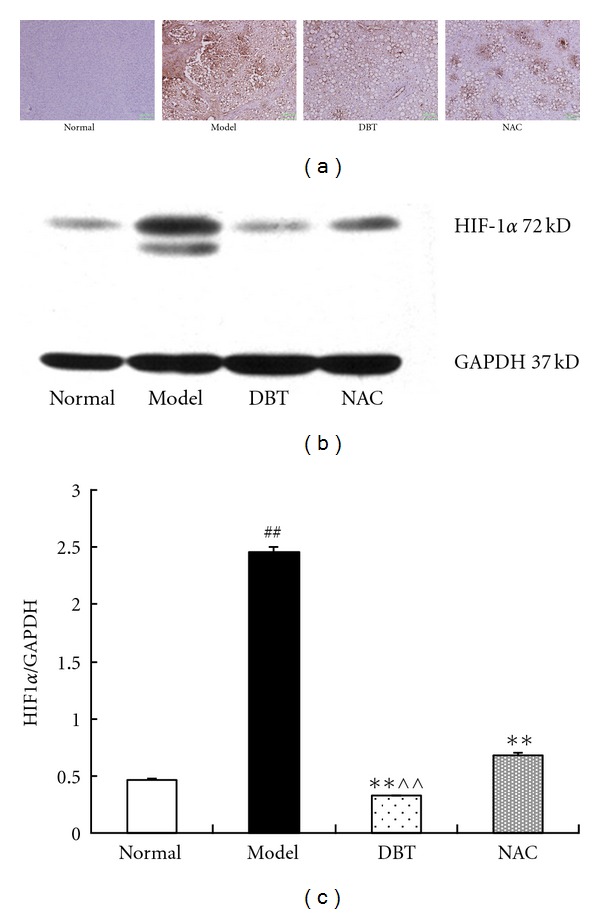
Effect of DBT and NAC on HIF-1*α* protein expression in CCl_4_-induced fibrotic liver tissue in rats. (a) Immunohistochemistry (DAB × 200). In normal livers, HIF-1*α* immunolabeling was rarely detected. In fibrotic livers, HIF-1*α* immunolabeling was highly expressed, especially in zone III of the acinus. DBT and NAC decreased HIF-1*α* expression in the fibrotic livers. (b) A representative western blot. Consistent with the immunohistochemistry findings, the semiquantitative value of the HIF-1*α* western blot was significantly increased in fibrotic livers. Compared to the model rats, DBT and NAC significantly decreased the expression of HIF-1*α*, with DBT being more effective than NAC. (c) A histogram showing HIF-1*α* protein semiquantified by densitometric analysis and expressed as the mean±SD for three separate experiments. ^##^ *P* < 0.01 versus normal group, **P < 0.01 versus model group, ^∧∧^ *P* < 0.01 versus NAC group.

Real-time PCR analysis showed that the expression of HIF-1*α* mRNA in the model livers was 3 times higher than in the normal group (Table 4). DBT and NAC significantly decreased this expression (Table 4). This was confirmed with HIF-1*α* immunostaining (Figure 4). In the livers of normal animals, HIF-1*α* immunolabeling was rarely detected. In the fibrotic livers, HIF-1*α* immunolabeling was highly expressed, especially in zone III of the acinus. DBT and NAC decreased HIF-1*α* expression in the fibrotic livers (*P* < 0.05). Consistent with the immunohistochemistry findings, western blot analysis showed that HIF-1*α* expression was significantly increased in the fibrotic livers (*P* < 0.01, versusnormal group). DBT and NAC decreased HIF-1*α* expression (*P* < 0.01), with a significant difference between the two treatment groups (*P* < 0.01).

### 3.5. Effects of DBT and NAC on Hepatic Oxidative Injury

CCl_4_ intoxication significantly increased hepatic contents of MDA and 8-OH-dG, and decreased hepatic SOD activity (Table 5) (*P* < 0.01, versus normal group). DBT and NAC reduced hepatic MDA and 8-OH-dG contents, increased the SOD activity (*P* < 0.05). In addition, DBT has better effect than NAC on increasing SOD activity in fibrotic liver.

### 3.6. Effects of DBT and NAC on Expression of VEGF, Ang1 and TGF-*β*1 and Their Signaling Mediators

**Figure 5:**
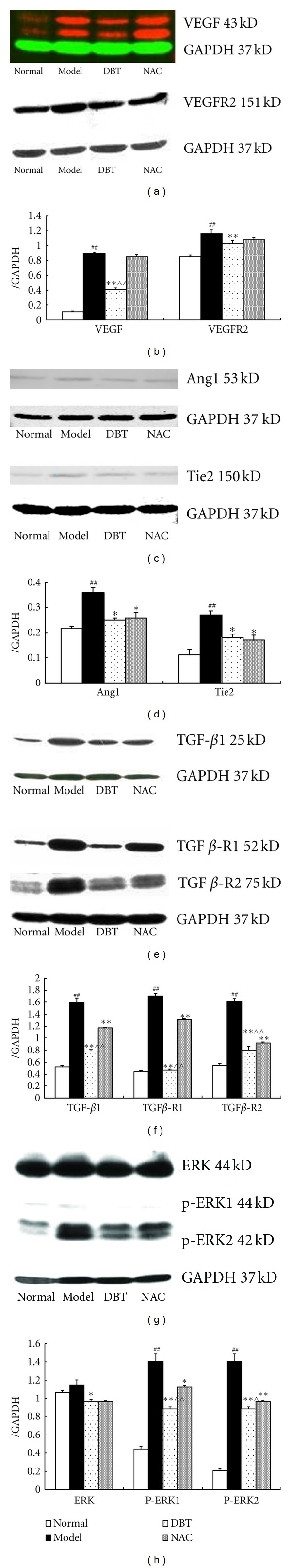
Effect of DBT and NAC on VEGF, Angiopoietin 1 and TGF-*β*1 signaling in CCl_4_-induced fibrotic liver tissue in rats. (a) The expression of VEGF and VEGFR2 protein was detected by western blot. Compared to the normal group, the expression of VEGF and VEGFR2 was significantly increased in fibrotic livers. DBT, but not NAC, significantly decreased VEGF and VEGFR2 expression, and there was a significant difference in VEGF expression between DBT- and NAC-treated groups. (c) The expression of the Angiopoietin 1 and Tie 2 protein was detected by western blot. Compared to the normal group, the expression of Ang1 and Tie 2 was increased in fibrotic livers. DBT and NAC decreased Ang1 and Tie 2 expression. (e) The expression of the TGF-*β*1/TGF*β*-R protein was detected by western blot. Compared to the normal group, TGF*β*1/TGF*β*-R protein expression was significantly increased in fibrotic livers. DBT and NAC significantly decreased the expression of TGF*β*1/TGF*β*-R. (g) The expression of ERK and p-ERK protein was detected by western blot. Although there was no significant difference in ERK protein expression among the four groups, p-ERK protein expression was significantly increased in fibrotic livers. DBT and NAC significantly decreased p-ERK protein expression, and there was a significant difference between DBT- and NAC-treated groups. (b, d, f, h) Histograms of VEGF/VEGFR2, Ang1/Tie 2, TGF*β*1/TGF*β*-R, and ERK/p-ERK expression. The proteins were semi-quantified by densitometric analysis and expressed as the mean ± SD for three separate experiments. ^##^ *P* < 0.01 versus normal group, **P < 0.01 versus model group, ^∧∧^ *P* < 0.01 versus NAC group.

Western blot analysis (Figure 5) showed that the expressions of pro-angiogenic growth factors such as VEGF, TGF-*β*1, and Ang1 elevated in fibrotic livers (*P *< 0.01, versus normal group), and their respective receptors—VEGF-R2, TGF*β*R-1/2 and Tie2 also increased. ERK is common down-stream signaling mediator for VEGF, TGF-*β*1, and so forth, the result showed that phosphorylation of ERK-1/2 increased remarkably, although total ERK1 has no change. DBT, but not NAC, significantly decreased VEGF and VEGF-R2 protein expression (*P* < 0.01), and there was a significant difference in VEGF expression between DBT- and NAC-treated groups. (*P* < 0.01). Both DBT and NAC down-regulated TGF*β*-R1/2 and Tie2 expression and ERK-1/2 phosphorylation in fibrotic liver (*P* < 0.05), compared to the model control. 

## 4. Discussion

The liver fibrosis, caused by many etiologies such as viruses, toxins, alcohol and cholestasis and so forth, is an essential pathological process in chronic liver diseases, and lead into cirrhosis which is end stage of chronic liver diseases. Therefore, it is very important to find an approach for curing or regressing liver fibrosis. Angiogenesis is the main process of new vessel formation, and accompanies liver fibrosis [2]. Recently a increasing body of evidences has shown that angiogenesis play a crucial role in liver fibrogenesis [14], and the inhibition of angiogenesis with multitargeted receptor tyrosine kinase inhibitors sunitinib or sorafenib could regress or reverse liver fibrosis in animals [15, 16]. Angiogenesis, a hypoxia-driven and growth factor-dependent process, also plays a key role in the development of cirrhosis [1, 2, 5, 17–20]. Since the conventional therapies are not desirable for curing liver fibrosis, the manipulation of angiogenesis could be a promising approach for treatment of liver fibrosis. 

According to traditional Chinese medicine (TCM) theory, liver fibrosis is caused by a dual deficiency of Qi-yin and blood stasis [21]. DBT has good function of nourishing Qi (energy flow) and Xue (blood), as well as resolving Yuxue (blood stasis), and has been used traditionally to treat menopausal disorders [6]. In our previous study, we found that DBT could ameliorate CCl_4_-induced liver fibrosis in rats [11]. In this study, we found that in normal livers, vWF and CD31, two markers of angiogenesis, are expressed few and limited to the large vessels of the periportal region and could not be found along sinusoids. However, their expression increased remarkably in fibrous septa and along sinusoids in fibrotic livers, which means that rich neovessels formed in fibrotic livers. While DBT and NAC treatment could decrease the expression of vWF and CD31 proteins, indicating that DBT and NAC can inhibit liver angiogenesis, which may be one of the action mechanisms of DBT and NAC antifibrotic property. It is reported recently that the thiol antioxidant NAC had effect against liver fibrosis [22] and decreased tumor angiogenesis and tumorigenesis [23]; our current study confirmed NAC's effect on liver fibrosis and find its new action on fibrotic liver angiogenesis. 

Angiogenesis is a dynamic, hypoxia-stimulated and growth factor-dependent process. A recent document has identified that there are number of common cellular and molecular mechanisms between angiogenesis and liver fibrosis, with a specific emphasis on the crucial role of hypoxic conditions, angiogenic factors and HSC activation. The hypoxia play a central role in angiogenesis through inducing HIF-1*α* [24]. HIF-1*α* is a heterodimeric DNA binding complex, it serve as a key regulator of the molecular hypoxia response and mediates a wide range of physiological and pathological processes. In the study, although we did not check hepatic hypoxia by assaying pO_2_ etc., HIF-1*α*, a hypoxia-driven factor, was mainly expressed in perivenous region (hepatic zone III area) where O_2_ concentration is lowest in liver and susceptible to be damaged. And fibrotic liver had much higher levels of gene and protein expression of HIF-1*α*, while DBT and NAC decrease this pathological elevated HIF-1*α* levels, in particular HIF-1*α* expression in perivenous region, these suggested that DBT and NAC action against liver angiogenesis was associated with down-regulation of HIF-1*α*, which at least partly own to improving hypoxia condition. 

However, HIF-1*α* upregulation could be hypoxia-independent, as elicited by growth factors, oncogenes, and oxidative stress and injury and so forth. The oxidative stress could cause lipid peroxidative products such as MDA and 8-OH-dG, leading to liver injury and angiogenesis [25], which can be regulated by endogenous antioxidant enzymes such as SOD and thioredoxin [26]. Recent studies have shown that antioxidants such as NAC can inhibit tumor angiogenesis [27]. In the study, we found DBT and NAC both improve SOD activity but decrease contents of MDA and 8-OH-dG in fibrotic liver, indicating that DBT and NAC inhibition of HIF-1*α* and angiogenesis is related to their effect on oxidative stress and injury. VEGF, TGF-*β*1, and Ang1 are potent angiogenic and fibrogenic factors, which may induce angiogenesis directly through their signaling pathway or indirectly by inducing HIF-1*α* which in turn promote its target genes including VEGF expression. VEGF, TGF-*β*1 and Ang1 stimulate vascular development and stabilization through signaling mediators, including their receptors—VEGF-R1/2 and TGF*β*-R1/2, Tie2 and mitogen-activated protein kinase (MAPK) cascade [28, 29]. In the present study, DBT not only inhibited the expression of growth factors including VEGF, TGF-*β*1, and Ang1, but also downregulated their receptors expression and ERK phosphorylation which is common cytoplasmic mediator, indicating that the inhibition of VEGF, TGF-*β*1 and Ang1 and their signaling is another important mechanism for DBT action on HIF-1*α*, and against angiogenesis and liver fibrosis.

In addition, MMP-2/9 play potent role in initiation of angiogenesis and activation of HSC, while activated HSCs synthesizing Ang 1 and VEGF contribute to both fibrogenesis and neovascularization [20]. In a previous study, we showed that the protective effect of DBT was associated with the inhibition of MMP 2/9 activities in fibrotic livers [11]. And the current experiment reconfirmed that DBT and NAC inhibited HSC activation *in vivo*, which suggest that inhibition of MMP-2/9 and HSC activation is the third mechanism of DBT action mechanism against angiogenesis in fibrotic liver. 

It is interesting to note that DBT can stimulate the production of erythropoietin, a specific hematopoietic growth factor, mediated by increasing the mRNA and protein expression of HIF-1*α* as well as the activation of Raf/MEK/ERK signaling pathway in cultured cells [30]. While these data appear to contradict our findings, these data collectively illustrate the potential of DBT and other herbal formulations in modulating cellular pathophysiology to achieve the desirable clinical outcome.

In summary, we found that DBT inhibits angiogenesis in CCl_4_-induced liver fibrosis in rats in the current study, which is closely related to DBT anti-fibrotic property; and that DBT reduces the expression of HIF-1*α*, VEGF, TGF-*β*1, and Ang1, decreases the receptors expression of VEGF-R2, TGF*β*-R1/2 and Tie2, downregulates ERK phosphorylation, and improves hepatic oxidative injury in fibrotic liver; these effects contribute to the overall action mechanisms of DBT against liver angiogenesis and fibrosis.

## Figures and Tables

**Table 1:** Antibodies used in the study.

Antibody	Isotype	Suppliers	Cat. no.	Dilution
Collagen type I	Mouse IgG1	Sigma	C2456	1 : 400
*α*-SMA	Rabbit polyclonal IgG	Abcam	ab5694	1 : 400
vWF	Rabbit polyclonal IgG	Abcam	ab6994	1 : 200
PECAM-1	Goat polyclonal IgG	Santa Cruz	sc-1506	1 : 200
HIF-1*α*	Rabbit monoclonal IgG	EPIT MICS	#2015-1	1 : 200
VEGF	Rabbit polyclonal IgG	Abcam	ab46154	1 : 400
VEGFR2	Rabbit polyclonal IgG	Abcam	ab39638	1 : 500
Angiopoietin 1	Rabbit polyclonal IgG	Abcam	ab95230	1 : 200
Tie-2	Rabbit polyclonal IgG	Abcam	ab71712	1 : 250
TGF-*β*1	Mouse IgG1	R&D systems	MAB240	1 : 500
TGF*β*-R1	Rabbit polyclonal IgG	Cell signal technology	#3712	1 : 1000
TGF*β*-R2	Mouse IgG_2a_	Santa Cruz	sc-17792	1 : 200
ERK	Mouse IgG_2b_	Santa Cruz	sc-1647	1 : 200
p-ERK	Mouse IgG_2a_	Santa Cruz	sc-7383	1 : 200

**Table 2:** The amounts of six compounds in DBT.

Compound	DBT (*Radix Astragali : Radix Angelica sinensis * = 5 : 1)
Astragaloside IV	61.63 ± 2.06
Calycosin	2.29 ± 0.08
Calycosin-7-O-*β*-D-glucoside	2.20 ± 0.03
Formononetin	0.68 ± 0.00
Formononetin-7-O-*β*-D-glucoside	1.12 ± 0.07
Ferulic acid	0.98 ± 0.02

Values are expressed in 10^−2^ mg/g of raw herbs and are in $\overset{-}{X}\pm \mathrm{sd}$, *n* = 3.

**Table 3:** Primers used for real-time PCR.

Gene	Primer sequences	Gene bank accession no.	Length (bp)
HIF1*α*	5^′^-CCAGATTCAAGATCAGCCAGCA-3^′^	NM_024359	100
	5^′^-GCTGTCCACATCAAAGCAGTACTCA-3^′^		
*β*-actin	5^′^-TGA CGA GGC CCA GAG CAA GA-3^′^	DQ237887	331
	5^′^-ATG GGC ACA GTG TGG GTG AC-3^′^		

**Table 4:** Effect of DBT on hepatic HIF-1*α* mRNA expression in CCl_4_-induced fibrosis in rats $\overset{-}{(X}\pm \mathrm{sd})$ *⁡*.

Group	*n*	HIF-1*α* /*β*-actin mRNA
Normal	3	0.98 ± 0.03
Model	3	3.32 ± 0.46^##^
DBT	3	0.49 ± 0.04^∗∗∧∧^
NAC	3	0.86 ± 0.05**

^##^ *P* < 0.01 versus normal group, **P < 0.01 versus model group, ^∧∧^ *P* < 0.01 versus NAC group.

**Table 5:** Effect of DBT and NAC on hepatic SOD activity and MDA and 8-OH-dG contents in CCl_4_-induced fibrosis ($\overset{-}{X}\pm \mathrm{sd}$).

Group	*n*	MDA (*μ*mol/g)	SOD (NU/g)	8-OHDG (ng/mL)
Normal	8	457.9 ± 140.6	42.9 ± 8.7	1.6 ± 0.6
Model	10	1054.5 ± 255.0^##^	24.7 ± 4.1^##^	2.8 ± 0.8^##^
DBT	12	642.8 ± 277.2**	37.7 ± 7.2^∗∗∧^	2.0 ± 0.4*
NAC	12	617.9 ± 344.5**	30.3 ± 15.8*	1.7 ± 0.9**

^##^ *P* < 0.01 versus normal group, **P < 0.01 versus model group, ^∧∧^ *P* < 0.01 versus NAC group. Two rats in model group died in 6 weeks of modeling.

## Glossary

DBT: Danggui Buxue Tang
NAC: N-Acetyl-L-cysteine
CCl_4_: Carbon tetrachloride
*α*-SMA: Alpha-smooth muscle actin
HIF-1*α*: Hypoxia inducible factor-1*α*
vWF: von Willebrand factor
PECAM-1: Platelet/endothelial cell adhesion molecule-1, also referred to as CD31
VEGF: Vascular endothelial growth factor
VEGF-R2: Vascular endothelial growth factor receptor 2, also referred to as KDR
TGF-*β*1: Transforming growth factor-*β*1
TGF*β*-R: Transforming growth factor-*β*1 receptor
p-ERK: Phospho-extracellular signal-regulated kinase
BSA: Bovine serum albumin
ECM: Extracellular matrix
CLDs: Chronic liver diseases
DAPI: 2-(4-Amidinophenyl)-6-indolecarbamidine dihydrochloride
Hyp: Hydroxyproline
HSC: Hepatic stellate cell
MMPs: Metalloproteinases
SOD: Superoxide dismutase
MDA: Malondialdehyde
8-0H-dG: 8-hydroxy-deoxyguanosine
Ang1: Angiopoietin 1
Tie2: Tyrosine kinase with immunoglobulin G-like and endothelial growth factor-like domain 2.
